# Fibroblasts Attenuate Anti-Tumor Drug Efficacy in Tumor Cells via Paracrine Interactions with Tumor Cells in 3D Plexiform Neurofibroma Cultures

**DOI:** 10.3390/cells14161276

**Published:** 2025-08-18

**Authors:** Kyungmin Ji, George J. Schwenkel

**Affiliations:** 1 Department of Neurology, Henry Ford Health, Detroit, MI 48202, USA; 2 Department of Pharmacology, Wayne State University, Detroit, MI 48201, USA

**Keywords:** plexiform neurofibromas, fibroblasts, drug resistance, 3D cultures

## Abstract

Plexiform neurofibromas (hereafter called pNF1) are often diagnosed in early childhood and occur in about 30% of neurofibromatosis type 1 (NF1) patients. pNF1 exhibits aggressive growth along a nerve in the body and has substantial potential for progression to malignant peripheral nerve sheath tumors that are rarely curable. There are two recently FDA-approved drugs, selumetinib and mirdametinib, for pNF1 patients who have symptomatic and inoperable plexiform neurofibromas; however, these treatments achieve only approximately 30% tumor shrinkage. Fibroblasts, the most abundant cell types within the pNF1 tumor microenvironment, are implicated in pNF1 growth and invasion; however, how fibroblasts affect a drug response of pNF1 remains poorly understood. In the present study, we focused on contributions of fibroblasts to the drug resistance in pNF1 via their secretome. We employed our established three-dimensional (3D) culture system incorporating human pNF1 tumor cells (*Nf1*^−/−^) and primary fibroblasts (*Nf1*^+/−^) grown in our patented microfluidic culture chips for monocultures and parallel cocultures in which 3D pNF1 structures and fibroblasts share their secretome without direct cell-to-cell contact. Three-dimensional pNF1 structures in 3D parallel cocultures with fibroblasts exhibited greater drug resistance than ones in monocultures. We found that pNF1 tumor cells showed increased P-glycoprotein expression when incubated with fibroblast-derived conditioned media or parallel cocultured with fibroblasts, compared to control conditions. Pharmacological inhibition of P-glycoprotein partially restored drug sensitivity. Additionally, fibroblasts showed higher resistance to selumetinib and mirdametinib than pNF1 tumor structures, likely due to elevated P-glycoprotein levels. This study is the first to define precise roles of fibroblasts in pNF1 drug resistance, emphasizing the potential of fibroblast-targeted therapies as a promising approach to improve pNF1 treatment outcomes.

## 1. Introduction

Neurofibromatosis type 1 (NF1) is caused by loss of expression of the neurofibromin (*Nf1* gene product) tumor suppressor gene, leading to dysregulated RAS/mitogen-activated protein kinase (MAPK) signaling in Schwann cells (SCs) (see reviews [1,2]). A hallmark of NF1 is the development of benign dermal neurofibromas (see reviews [1,2]). About 30% of children with NF1 develop plexiform neurofibromas (hereafter called pNF1) [3], which can cause significant morbidity due to their invasive growth and nerve compression [4,5]. pNF1 tumors have a 10–15% risk of progressing into malignant peripheral nerve sheath tumors (MPNSTs), which are aggressive and often fatal [6]. There are two FDA-approved drugs, selumetinib (AZD6244) [7,8] and mirdametinib (PD0325901) [9,10], for pNF1 patients with symptomatic and inoperable tumors [11,12]. However, these treatments have demonstrated only partial tumor shrinkage of ~30% [11,12], highlighting the need for new and more effective strategies to enhance their efficacy.

pNF1 tumors consist of SC-derived tumor cells (*Nf1*^−/−^) and the tumor microenvironment (TME), including cellular (e.g., fibroblasts, endothelial cells, and inflammatory cells; *Nf1*^+/−^) and patho-chemical components resulting from hypoxia and acidosis [13]. The cellular microenvironment is implicated in the development of neurofibromas through a paracrine loop between the tumor cells and the cellular microenvironment [13,14,15], yet its impact on drug response of the pNF1 remains unclear. Fibroblasts, the major cell types in the pNF1 TME [16,17], produce a dense collagenous extracellular matrix (ECM), a hallmark of neurofibromas [18,19], and facilitate tumorigenesis of pNF1 [20]. Transforming growth factor beta (TGF)-β produced by mast cells increase the recruitment to tumor regions and collagen synthesis of fibroblasts during pNF1 progression [21]; however, how paracrine interaction of fibroblasts and other cells in tumor niche affects pNF1 growth and drug response remains underexplored. Using our unique three-dimensional (3D)/4D (3D + real-time) culture models, we have previously reported that secretory factors, i.e., the secretome, from fibroblasts enhance the growth and invasiveness of 3D pNF1 tumor structures as seen in tumors treated with fibroblast-conditioned media (CM) compared to control media [22]. A deeper understanding of fibroblast-mediated drug resistance in pNF1 is urgently needed to develop effective therapeutic strategies.

To our knowledge, there are no studies or 3D culture-based pre-clinical platforms related to the evaluation of drug resistance in pNF1 tumor cells within the context of their TME. In the present study, we investigated the paracrine effects of the fibroblast-derived secretome on drug responses in 3D pNF1 tumor structures formed by human immortalized pNF1 cell lines, ipNF95.11bC and ipNF05.5, treated with either selumetinib or mirdametinib. Our data demonstrate that fibroblasts enhance drug resistance in 3D pNF1 tumor structures via their secretome, suggesting that therapies targeting fibroblasts may improve the efficacy of selumetinib or mirdametinib in pNF1 patients.

## 2. Materials and Methods

### 2.1. Reagents and Antibodies

Reconstituted basement membrane (rBM; Cultrex^TM^ with reduced growth factor) and high-concentration collagen I (rat tail) were purchased from Bio-Techne (Minneapolis, MN, USA) and Corning (Corning, NY, USA), respectively. Phenol red-free Dulbecco’s Modified Eagle Medium (DMEM) and MycoZap^TM^ Plus-CL were purchased from Lonza (Basel, Switzerland). Fetal bovine serum (FBS) was obtained from Cytiva (Marlborough, MA, USA). Selumetinib (AZD6244), mirdametinib (PD0325901), and tariquidar (XR9576) were purchased from Selleckchem (Houston, TX, USA). L-glutamine and all other chemicals unless otherwise stated were purchased from Sigma-Aldrich (St. Louis, MO, USA). Fluorescent dyes and LIVE/DEAD^TM^ Viability/Cytotoxicity kit, Hoechst33342, and 4′-6-Diamidino-2-phenylindole (DAPI), were purchased from ThermoFisher Scientific (Waltham, MA, USA). Anti-P-glycoprotein primary antibody and Cy3-conjugated secondary antibody were purchased from Abcam (Cambridge, UK; ab170904) and Jackson ImmunoResearch (West Grove, PA, USA), respectively.

### 2.2. Cells and Cell Maintenance

The following immortalized human plexiform neurofibroma cell lines (*Nf1*^−/−^; hereafter called pNF1 cells) described in [23] were used: ipNF95.11b C and ipNF05.5 purchased from ATCC. Primary fibroblasts (*Nf1*^+/−^, hereafter called fibroblasts) from pNF1 patients were purchased from Dr. Margaret Wallace, University of Florida (Gainesville, FL, USA). Cells were maintained as monolayers at 37 °C, 5% CO_2_ in DMEM/high glucose supplemented with 10% FBS. Routine screening ensured the absence of mycoplasma contamination.

### 2.3. Three-Dimensional (3D)/4D (3D in Real-Time) Culture Models

We employed 3D cultures grown in the patented microfluidic culture devices called TAME (tissue architecture and microenvironment engineering; Patent# US10227556B2) chips [24] for all experiments in the present study as previously reported [22,23]. Details for fabrication and prototypes of TAME chips with separate or linked wells [24] and the protocols for 3D monocultures and 3D parallel cocultures [25] were described in our previous report. For 3D monocultures of pNF1 cells and fibroblasts, cells were seeded on rBM, Cultrex^TM^/collagen I mixture, on the glass bottom culture well of the TAME chips with separate wells overlaid with 2% rBM in DMEM and grown for 5 days. For 3D pNF1 cell/fibroblast parallel cocultures, pNF1 cells and fibroblasts were plated on top of rBM in each well of TAME chips with linked wells, followed by 2% rBM in DMEM. A seeding ratio of 1 pNF1 tumor cell to ½ or ¼ fibroblast was used for 3D parallel cocultures. Prior to 3D live-cell imaging, nuclei were stained with Hoechst33342 to assess cell conditions by visualizing the integrity and morphology of the cell nuclei and to quantify total live-cell number with live-cell markers (e.g., Calcein AM).

### 2.4. Image Acquisition for Quantitative Analysis in 3D

Optical sections spanning the full depth of the 3D structures were acquired from four contiguous fields (2 × 2) using either an upright Zeiss LSM 780 confocal microscope (Carl Zeiss Microscopy, Jena, Germany) housed within a temperature- and CO_2_-controlled environmental chamber or an inverted Leica Stellaris 5 system (Leica Microsystems, Deerfield, IL, USA) equipped with a Tokai Hit stage-top incubator (Tokai Hit, Shizuoka, Japan) to maintain physiological conditions. Image stacks were processed and reconstructed in 3D using Volocity software (Version 7.0.0; PerkinElmer, Waltham, MA, USA) for visualization and quantification of live/dead cell distributions. The spatial orientation within reconstructed volumes is indicated by x (green), y (red), and z (blue) arrows displayed in the lower corner of each image.

### 2.5. Drug Treatments

Cells were seeded and cultured for 1 day to allow the settlement of cells in 3D rBM. On day 1, media were replaced, and drugs, including selumetinib [7,8] (10 μM) or mirdametinib [9,10] (1 μM), the only FDA-approved drugs for pNF1 treatment, and/or XR9576 [26,27] (0.1 μM), a specific P-glycoprotein inhibitor, were treated in 3D cell cultures for 4 days.

### 2.6. Live-Cell LIVE/DEAD^TM^ Assay

3D cultures were incubated with Calcein AM (live cells, green) for 30 min at 37 °C and washed once with warm PBS, then warm culture media were added according to the manufacturer’s recommended protocols and previously published reports [28,29,30]. Nuclei were stained with Hoechst33342 prior to live-cell imaging to confirm the cell conditions such as nuclear condensation and to quantify live cell number accurately. Cells were imaged live on the confocal microscopes. The number of live cells was calculated by counting the number of intact and healthy nuclei (appearing as evenly stained and spherical, not condensed and fragmented) overlapped with Calcein AM-stained cells in 3D using Volocity.

### 2.7. Generation of Fibroblast-Conditioned Media (CM)

Details for generation of Fibroblast-CM were described in our previous report [22]. Briefly, fibroblasts (1 × 10^6^ cells in a 60 mm culture dish) were cultured in fresh DMEM for 5 days. Fibroblast-conditioned media (Fib-CM) were collected and centrifuged at 1800× *g* to remove debris, and the resulting supernatants were aliquoted to minimize freeze–thaw cycles. Both Fib-CM and cell-free control media (CtrlM) were stored at −20 °C until use.

### 2.8. Immunofluorescence Staining

We adapted the Sloane group’s immunostaining method for 3D cultures [29]. Cells were cultured for 5 days in 3D cultures as monocultures, pNF1:Fibroblast parallel cocultures, or in media supplemented with CtrlM or Fib-CM. Following incubation, they were fixed with 10% formaldehyde for 10 min at room temperature (RT), washed with warm PBS, and permeabilized for 5 min using 0.2% Triton X-100 in warm PBS. The cells were blocked with 1% BSA for an hour at RT, incubated with primary antibody (P-glycoprotein) for 3 h at RT, then treated with fluorescent secondary antibody for an hour at RT. DAPI was used for nuclear staining. Images were captured using an inverted Stellaris 5 confocal microscope (Leica Microsystems). Fluorescence intensity of P-glycoprotein was quantified using ImageJ 1.54g software (National Institutes of Health, Bethesda, MD, USA).

### 2.9. 3D MTT Assay

3D MTT assay was used to assess cell growth and viability as previously described [22]. Briefly, 1.2 × 10^4^ pNF1 cells were seeded in 150 μL media of DMEM supplemented with 10% FBS on 3D rBM, with an additional 2% rBM overlay per well (triplicate or quadruplicate). On day 1, the media were replaced with fresh DMEM (3% FBS), and designated drug treatments were applied. After 4 days of drug treatments (day 5), MTT solution was added, and cells were incubated for 3 h to allow formazan formation. The resulting crystals were solubilized overnight at 37 °C in a solution of 10% SDS and 0.01% HCl, facilitating extraction from both the cells and surrounding rBM. Absorbance was measured at 570 nm with background correction at 690 nm using a SpectraMax Plus 384 microplate reader (Molecular Devices, San Jose, CA, USA).

### 2.10. Statistical Analysis

Data are presented as bar graphs with individual data points, representing the mean ± standard deviation (SD) from at least three independent experiments. The number of replicates is provided in each corresponding figure legend. Statistical significance was assessed using either one-way ANOVA followed by Tukey’s post hoc test for multiple group comparisons or Student’s t-test for comparisons between two groups. A *p*-value ≤ 0.05 was considered statistically significant throughout the study.

## 3. Results

### 3.1. Paracrine Interactions Between 3D pNF1 Tumor Structures and Fibroblasts Increase Drug Resistance in 3D pNF1 Culture Model

**Figure 1:**
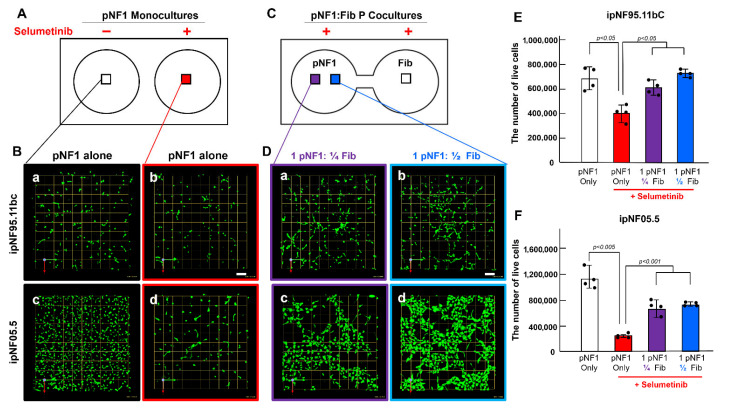
**Fibroblast-derived secretome increases selumetinib-induced drug resistance in 3D pNF1 structures.** (**A**,**C**) Schematics of the TAME chips with separate wells for 3D pNF1 monocultures (**A**) or linked wells for 3D pNF1:fibroblast (Fib) parallel cocultures (**C**; P cocultures). Boxes in each well indicate where the images in (**B**,**D**) were taken. (**B**,**D**) En face views of 3D reconstructions of 3D pNF1 structures formed by ipNF95.11bC [**B**, top row (**a**,**b**)] and ipNF05.5 [**B**, bottom (**c**,**d**)] in monocultures without [**B**, left panel (**a,c**)] or with selumetinib [**B**, right (**b**,**d**)] and in parallel cocultures with fibroblasts at different ratios (**D**) with selumetinib [ipNF95.11bC (**D**(**a**,**b**)); ipNF05.5 (**D**(**c**,**d**))]. Green signals indicate live cells stained by Calcein AM. Different ratios of pNF1 cells and fibroblasts at 1:¼ [**D**, left panel (**a**,**c**)] or 1:½ [**D**, right (**b**,**d**)] were used. Images are tiled from 4 contiguous fields; grid and scale bars, 174 μm. (**E**,**F**) Total number of live pNF1 tumor cells [ipNF95.11bC (**E**) and ipNF05.5 (**F**)] in monocultures (white bars, no treatment; red, selumetinib-treated) and parallel cocultures (purple, selumetinib-treated 1 pNF to ¼ Fib parallel coculture; blue, selumetinib-treated 1 pNF to ½ Fib parallel coculture) was quantified in 3D using Volocity. Data are expressed as mean ± standard deviation (SD) (*n* = 4).

To investigate paracrine effects of fibroblasts on drug resistance in 3D pNF1 tumor structures, formed by human immortalized pNF1 cell lines, ipNF9511bC or ipNF05.5, we employed our 3D cultures grown in the patented microfluidic culture chips called TAME chips [22,24]. Cells were cultures in the TAME chips either with separate wells (illustrated in Figure 1A) for 3D pNF1 monocultures [Figure 1B, ipNF95.11bC (top; a,b) and ipNF05.5 (bottom; c,d)] or with linked wells (illustrated in Figure 1C) for 3D pNF1:fibroblast parallel cocultures [Figure 1D, ipNF95.11bC (top; a,b) and ipNF05.5 (bottom; c,d)]. In the linked-well configuration, the two cell types remained physically separated while sharing their secretome. Since a recent study has shown that fibroblasts account for a significant portion of the tumor volume, about 60% in pNF1 patients [17], we used a ratio of 1 tumor to ¼ (Figure 1D left panel; a,c) or ½ fibroblast (Figure 1D right; b,d) for 3D pNF1:fibroblast parallel cocultures. We compared drug responses in 3D pNF1 tumor structures grown as monocultures and as parallel cocultures with fibroblasts with 4-day selumetinib treatment using the Calcein AM of LIVE/DEAD^TM^ Viability/Cytotoxicity kit. In pNF1 monocultures, the number of live 3D pNF1 structures was reduced by about 40% (ipNF95.11bC) and 50% (ipNF05.5) with 4-day selumetinib treatment (Figure 1B,E,F). In 3D pNF1:fibroblast parallel cocultures (Figure 1D), the number of live 3D pNF1 structures was significantly greater than in monocultures (Figure 1B(b,d)) with the same 4-day selumetinib treatment, which was verified by 3D quantitative analysis using Volocity [Figure 1E (ipNF95.11bC) and Figure 1F (ipNF05.5)].

**Figure 2:**
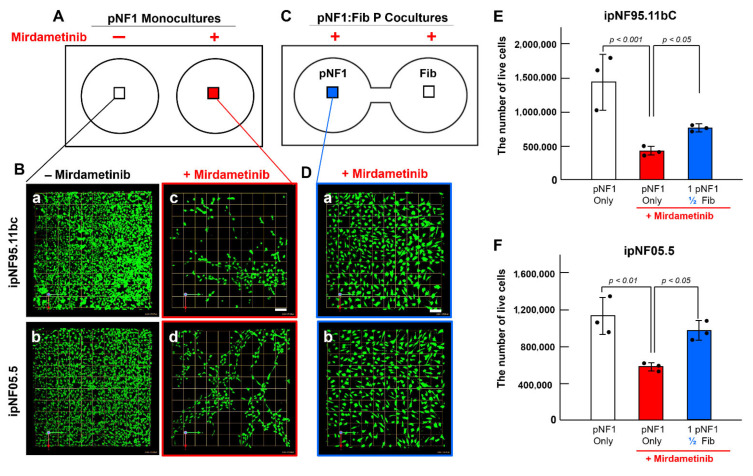
**Fibroblast-derived secretome also promotes drug resistance in pNF1 tumor structures treated with mirdametinib.** (**A**,**C**) Schematics of the TAME chips with separate wells for 3D pNF1 monocultures (**A**) or linked wells for 3D pNF1:Fib parallel cocultures (**C**; P cocultures) with and without mirdametinib. Boxes in each well indicate where the images in (**B**,**D**) were taken. (**B**,**D**) En face views of 3D reconstructions of pNF1 structures formed by ipNF95.11bC [**B**, top row (**a**,**c**)] and ipNF05.5 [**B**, bottom (**b**,**d**)] in monocultures without [**B**, left panel (**a**,**b**)] or with mirdametinib [**B**, right (**c**,**d**)], and in parallel cocultures with fibroblasts at a ratio of 1 tumor to ½ fibroblasts with mirdametinib [ipNF95.11bC (**D**(**a**)), ipNF05.5 (**D**(**b**))]. Green signals indicate live cells stained by Calcein AM. Images are tiled from 4 contiguous fields; grid and scale bars, 174 μm. (**E**,**F**) Total number of live pNF1 cells [ipNF95.11bC (**E**) and ipNF05.5 (**F**)] in monocultures (white bars, no treatment; red, mirdametinib-treated) and parallel cocultures (blue, mirdametinib-treated) was quantified in 3D using Volocity. Data are expressed as mean ± SD (*n* = 3).

To determine whether the fibroblast-derived secretome has similar effects on drug resistance in pNF1 tumor cells when treated with the other FDA-approved drug for pNF1 patients, we assessed the drug response of 3D pNF1 tumor structures to mirdametinib (PD0325901) in monocultures (Figure 2A,B) and parallel cocultures with fibroblasts at a ratio of 1 tumor to ½ fibroblasts (Figure 2C,D) over the same treatment period. Similarly, the number of live tumor structures was higher in parallel cocultures than in monocultures [Figure 2E (ipNF95.11bC) and Figure 2F (ipNF05.5)].

Since our previous report has shown that fibroblast-derived CM increases the growth of pNF1 structures in 3D cultures without drug treatment [22], we investigated whether the drug resistance in 3D pNF1 structures observed in pNF1:fibroblast parallel cocultures with drug treatment could be caused by enhanced tumor cell growth from fibroblast-derived secretome. We grew pNF1 cells, ipNF95.11bC and ipNF05.5, in monocultures and in parallel cocultures at different pNF1:fibroblast ratios without drug treatment over a 5-day culture period. We observed a minimal increase in the growth of tumor structures between pNF1 monocultures and pNF1:fibroblast parallel cocultures (Supplemental Figure S1). These results clearly indicate that the increased drug resistance of pNF1 tumor structures with the treatment of selumetinib or mirdametinib is driven by tumor:fibroblast paracrine interactions, rather than by fibroblast secretome-induced tumor growth alone.

### 3.2. Fibroblasts Show Greater Resistance than pNF1 Tumor Structures to the Treatment of Selumetinib or Mirdametinib in 3D Cultures

**Figure 3:**
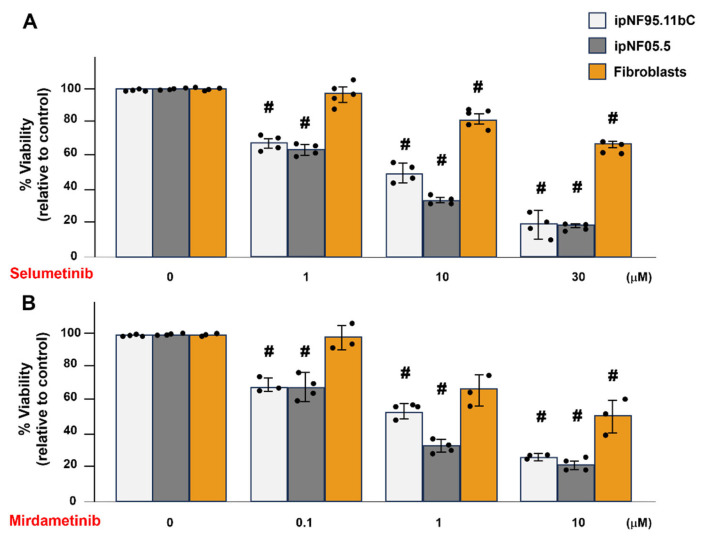
**Fibroblasts exhibit reduced sensitivity compared to pNF1 tumor structures with 4-day selumetinib or mirdametinib treatment in 3D culture.** 3D monocultures of pNF1 tumor cells [ipNF95.11bC (white bars) and ipNF05.5 (gray)] and fibroblasts (orange) were treated with selumetinib (**A**) or mirdametinib (**B**) at the indicated concentrations for 4 days. Cell viability was measured using a 3D MTT assay and expressed as a percentage of control groups (no drug treatment). Data represent mean ± SD (*n* = 3–5). # *p* < 0.05 vs. control groups (no drugs).

Selumetinib and mirdametinib have been used to shrink tumors in pNF1 patients [11,12]; however, to our knowledge, there are no studies on how these drugs affect the pNF1 TME. Our earlier findings showed that fibroblast-derived secretome increases pNF1 tumor growth and invasion [22], and drug resistance, therefore we sought to determine the sensitivity of fibroblasts in the pNF1 TME to these drugs. Using a 3D MTT assay, we compared drug sensitivity of fibroblasts and tumor structures with different dosages of selumetinib (Figure 3A) or mirdametinib (Figure 3B) for 4 days. Surprisingly, fibroblasts were less sensitive to these drugs than 3D pNF1 tumor structures in 3D cultures. These results suggest that fibroblast-targeted treatments are urgently needed to improve the therapeutic outcome of selumetinib and mirdametinib treatment for pNF1 patients.

### 3.3. P-Glycoprotein Contributes to Fibroblast-Derived Secretome-Mediated Drug Resistance in pNF1 Tumor Structures

**Figure 4:**
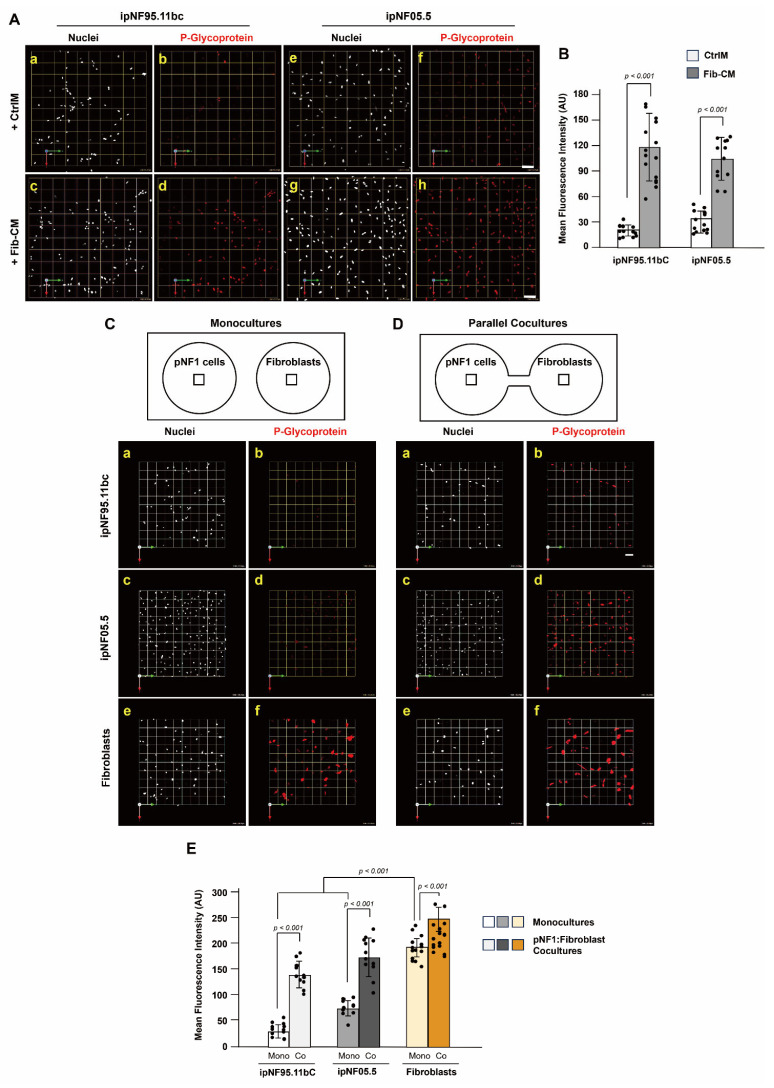
**Fibroblasts enhance Pgp expression in pNF1 tumor structures and show higher levels themselves.** (**A**) En face views of 3D reconstructions of pNF1 structures [ipNF95.11bC (**a**–**d**) and ipNF05.5 (**e**–**h**)] in 3D pNF1 monocultures incubated with CtrlM (top; **a**,**b**,**e**,**f**) fibroblast-derived CM (bottom; **c**,**d**,**g**,**h**) for 5 days. Cells were stained with Pgp (red) and Hoechst33342 (nuclei; pseudo-white). Grid and scale bars indicate 91 μm. (**B**) Relative mean fluorescence intensity of Pgp in pNF1 tumor cell lines cultured with control media (CtrlM; white bars) or fibroblast-derived conditioned media (Fib-CM; gray bars) was quantified using ImageJ. Data represent mean ± SD (*n* = 3–4). (**C**,**D**) En face views of 3D reconstructions of pNF1 structures [ipNF95.11bC (**C**(**a**,**b**) and **D**(**a**,**b**)) and ipNF05.5 (**C**(**c**,**d**) and **D**(**c**,**d**))] and fibroblasts (**C**(**e**,**f**) and **D**(**e**,**f**)) in 3D monocultures (**C**) or parallel cocultures (**D**) for 5 days. Cells were stained with Pgp (red) and Hoechst33342 (nuclei; pseudo-white). Grid and scale bars indicate 91 μm. (**E**) Relative mean fluorescence intensity of Pgp in pNF1 tumor cells and fibroblasts under monocultures (Mono) or parallel pNF1:fibroblast cocultures (Co; tumor-to-fibroblast ratio of 1:1/2) was quantified using ImageJ. Data represent mean ± SD (*n* = 3–4).

Our next question is how fibroblasts in the pNF1 TME increase drug resistance in pNF1 tumor cells through their secretome in 3D pNF1 culture models. P-glycoprotein [Pgp; also known as multi-drug resistance protein (MDR)1, ATP-binding cassette sub-family B member 1 (ABCB1), or CD243], is a drug efflux transporter leading to reduced efficacy of chemotherapy in tumor cells (see reviews [31,32,33,34]). We first assessed whether the fibroblast-derived secretome affects the expression level of Pgp in 3D pNF1 tumor structures. pNF1 tumor cells treated with fibroblast-derived conditioned media (Fib-CM; Figure 4A(c–h),B) exhibited significantly higher Pgp levels than those treated in control media (CtrlM; Figure 4A(a–f),B). Similarly, pNF1 tumor cells in parallel cocultures with fibroblasts at a 1 tumor to ½ fibroblast ratio (Figure 4D(a–d),E) showed elevated Pgp expression compared to monocultures (Figure 4C(a–d),E). Notably, fibroblasts in 3D cultures displayed even greater Pgp expression than pNF1 tumor structures (Figure 4C(e,f),D(e,f),E), correlating with their increased drug resistance demonstrated in Figure 3. These results indicate that fibroblast-derived paracrine signaling and the secretome promote drug resistance in pNF1 tumor structures by upregulating Pgp, with fibroblasts themselves exhibiting even higher expression levels consistent with their greater resistance in 3D cultures.

**Figure 5:**
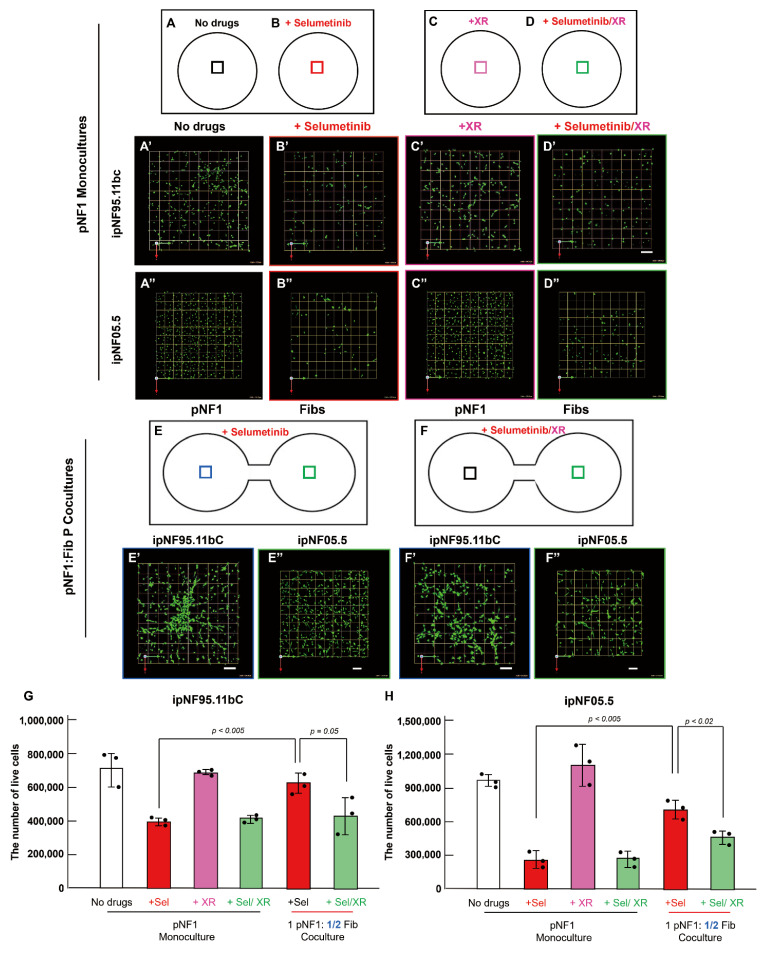
XR9576, a specific Pgp inhibitor, rescues drug resistance in pNF1 structures mediated by the secretome released from fibroblasts. (**A**–**F**) Schematics of the TAME chips with separate wells for 3D pNF1 monocultures (**A**–**D**) or linked wells for 3D pNF1:fib parallel cocultures (**E**,**F**) with no drugs (**A**), selumetinib (**B**,**E**), XR5976 (XR; **C**), selumetinib and XR (**D**,**F**). Boxes in each well indicate where the images in (**A′**–**F″**) were taken. (**A′**–**F″**) En face views of 3D reconstructions of pNF1 structures formed by ipNF95.11bC (**A′**–**F′**) or ipNF05.5 (**A″**–**F″**) in 3D monocultures (**A′**–**D″**) and parallel cocultures with fibroblasts at a ratio of 1 tumor to ½ fibroblasts (**E′**–**F″**) with the corresponding drugs in (**A**–**F**). Green signals indicate live cells stained by Calcein AM. Images are tiled from 4 contiguous fields; grid and scale bars, 174 μm. (**G**,**H**) Total number of live pNF1 tumor cells [ipNF95.11bC (**G**) and ipNF05.5 (**H**)] was quantified in 3D using Volocity (*n* = 3).

To test whether XR9576, a potent and specific Pgp inhibitor, could reverse fibroblast-induced drug resistance, we compared its effects in 3D pNF1 tumor structures grown in monoculture and parallel cocultures with fibroblasts. 3D pNF1 tumor structures formed by ipNF95.11bC (A′–F′) or ipNF05.5 cells (A″–F″) were cultured either alone (A–D) or with fibroblasts at a 1:½ tumor-to-fibroblast ratio (E,F) for 5 days. In pNF1 monocultures, selumetinib significantly reduced viable pNF1 tumor structures (Figure 5A–A″ vs. Figure 5B–B″), whereas XR9576 alone (Figure 5C–C″) or combined with selumetinib (Figure 5D–D″) did not impact tumor viability under these conditions. In contrast, in pNF1:fibroblast parallel cocultures, XR9576 successfully rescued selumetinib’s efficacy by overcoming fibroblast-derived drug resistance (Figure 5E–E″ vs. Figure 5F–F″), as confirmed by 3D quantification (Figure 5G,H). Taken together, XR9576 effectively rescued fibroblast-mediated resistance to selumetinib in 3D pNF1 tumor structures.

## 4. Discussion

Fibroblasts are the most abundant cells in the pNF1 TME, yet their precise roles in pNF1 progression have been under-investigated. Our previous report has demonstrated the critical role of fibroblasts in promoting the growth and invasive character of pNF1 tumor structures via their secretome in our 3D pNF1 culture models [22]. In the present study, we found that fibroblasts enhance drug resistance in 3D pNF1 tumor structures via paracrine interactions, with Pgp potentially mediating this resistance. Moreover, fibroblasts exhibit greater drug resistance than pNF1 tumor structures to selumetinib and mirdametinib, the only FDA-approved drugs for pNF1. These results highlight the importance of investigating fibroblast driven drug resistance and emphasize the urgency of targeting fibroblast function or blocking paracrine signaling between fibroblasts and tumor cells to improve treatment outcomes for pNF1 patients. Additionally, our 3D culture models integrated with the patented microfluidic culture chips provide a reliable pre-clinical model for high-content live-cell imaging and semi-high-throughput assays on pNF1 growth and drug screening/development.

Fibroblasts regulate drug resistance of cancer cells by several mechanisms, including (1) metabolic reprogramming, ECM remodeling, and immune suppression by interacting with the TME and (2) increasing drug efflux and epithelial–mesenchymal transition (EMT) induction by interacting with tumor cells (see reviews [35,36,37,38]). Although many studies have investigated therapeutics that target fibroblasts using combination strategies to enhance the effectiveness of anti-cancer drugs and block fibroblast interactions with the TME, these trials have shown disappointing outcomes [35,38,39]. A deeper understanding of the heterogeneous functionality of fibroblasts is needed to elucidate the paracrine interactions of fibroblasts, the TME, and tumor cells.

A recent study reported that pNF1 tumors consist of approximately 5.9% tumor cells and 62.6% fibroblasts, indicating a 1:10 tumor-to-fibroblast ratio [17]. Our previous work demonstrated that even a modest fibroblast proportion (1:¼) in direct 3D cocultures significantly promotes pNF1 tumor structure growth [22]. In this study, we show that low fibroblast ratios (1:½ and 1:¼) similarly increase resistance to selumetinib and mirdametinib in 3D pNF1 parallel cocultures, where tumor cells and fibroblasts are physically separated within microfluidic chips. The absence of a statistically significant difference between these ratios suggests a threshold effect, potentially driven by paracrine signaling saturation, spatial limitations, or diffusion constraints in the 3D extracellular matrix. Additionally, cytokine array analysis of 3D fibroblast monocultures revealed high levels of interleukin (IL)-6, monocyte chemoattractant protein (MCP)-1, IL-8, and tissue inhibitor of metalloproteinases (TIMP)-2 along with evidence suggesting small extracellular vesicles (sEVs) derived from fibroblasts may further promote the growth and invasiveness of tumor cells in 3D pNF1 cultures [22]. Further investigation into the fibroblast secretome is warranted as identifying its druggable pathways may lead to more effective strategies for overcoming tumor drug resistance.

Multidrug resistance (MDR) in tumor cells is mediated by genetic or epigenetic changes (see reviews [40,41,42]) and is one of the major reasons for chemotherapy failure. MDR is associated with various mechanisms, including increased drug efflux, alteration of the drug target, enhanced DNA damage recovery, inhibition of cell death, and interactions with the TME [42,43]. Pgp is a protein involved in MDR in tumors and is an ATP-dependent drug-efflux pump that decreases intracellular drug accumulation (see reviews [31,32,33,34]). Several pre-clinical studies using Pgp inhibitors have shown a reversal or delay of drug resistance in ovarian and prostate [44,45], colon [46,47,48], and non-small-cell lung cancer cells [45]. However, efforts to reduce drug resistance using Pgp inhibitors have had limited efficacy due to their clinical toxicity from their broad substrate specificity, pharmacokinetic interactions with other drug transporters and enzymes, and constitutive expressions in both healthy and tumor cells [31]. Therefore, the development of more potent and selective drugs targeting specific pathways to prevent drug resistance with minimal toxicity is essential to improving the treatment of pNF1 patients.

There are a number of caveats to the present study. Although our 3D/4D culture model offers valuable insights, it only partially reflects the *in vivo* complexity of pNF1, particularly the cellular diversity of neurofibromas, fibroblast heterogeneity, and the roles of other cell types such as T cells [49] and neurons [50]. To better understand the tumor microenvironment (TME) of pNF1, future studies should incorporate additional cellular components. We observed a partial reversal of drug resistance in 3D pNF1 tumor structures following treatment with XR9576, a specific Pgp inhibitor. As next steps, we plan to (1) confirm the functional role and activity of Pgp in the fibroblast secretome-mediated drug resistance in pNF1 monocultures vs. parallel cocultures with fibroblasts; (2) investigate the mechanism by which the fibroblast-derived secretome regulates Pgp expression, either through extracellular vesicle-mediated transfer or via secretome-induced expression; and (3) assess drug resistance following Pgp knockdown in pNF1 tumor cells. Other MDR mechanisms along with other alternative pathways that can regulate drug resistance in pNF1 tumor cells need to be examined. Additionally, we aim to determine whether the fibroblast-derived secretome antagonizes MEK pathway inhibition induced by the FDA-approved MEK inhibitors, selumetinib and mirdametinib. Finally, our findings highlight the need for pNF1 murine model studies to assess Pgp inhibitors in combination with selumetinib or mirdametinib for long-term drug efficacy.

## 5. Conclusions

Our findings reveal the crucial role of the fibroblast-derived secretome in promoting drug resistance in 3D pNF1 tumor structures through paracrine interactions. Using our 3D culture models with the patented microfluidic TAME chips, we observed that fibroblasts enhance tumor survival under selumetinib or mirdametinib treatment conditions. This increased resistance is associated with elevated P-glycoprotein expression in both tumor structures and fibroblasts, suggesting a mechanism by which the fibroblast-derived secretome contributes to reduced drug efficacy. Furthermore, fibroblasts demonstrated greater resilience to selumetinib or mirdametinib treatment compared to tumor structures, underscoring the urgent need for fibroblast-targeted therapies to improve treatment outcomes. Notably, inhibition of P-glycoprotein using XR9576 successfully rescued drug resistance in tumor structures within fibroblast cocultures, supporting its potential as a therapeutic strategy. These findings provide valuable insights into the tumor microenvironment and emphasize the importance of targeting fibroblast-driven resistance in pNF1 treatment.

## 6. Patents

The present study is associated with the U.S. Patent US10227556B2.

## Data Availability

The data presented in this study are openly available from the MDPI website or upon request from the corresponding author. [zenodo] [10.5281/zenodo.15270440].

## Supplementary Materials

The following supporting information can be downloaded at: https://www.mdpi.com/article/10.3390/cells14161276/s1, Supplemental Figure S1: Fibroblast-derived secretome induces a minimal increase in pNF1 tumor structure growth in 3D pNF1 cell:fibroblast parallel cocultures.
