# Supplementary material for: Fibroblasts Attenuate Anti-Tumor Drug Efficacy in Tumor Cells via Paracrine Interactions with Tumor Cells in 3D Plexiform Neurofibroma Cultures
Source: Cells. 2025 Aug 18;14(16):1276. doi: 10.3390/cells14161276 (PMC12384539; doi:10.3390/cells14161276)
Supplement: Supplementary file 1 [file cells-14-01276-s001.zip › cells-3633554-supplementary.pdf]

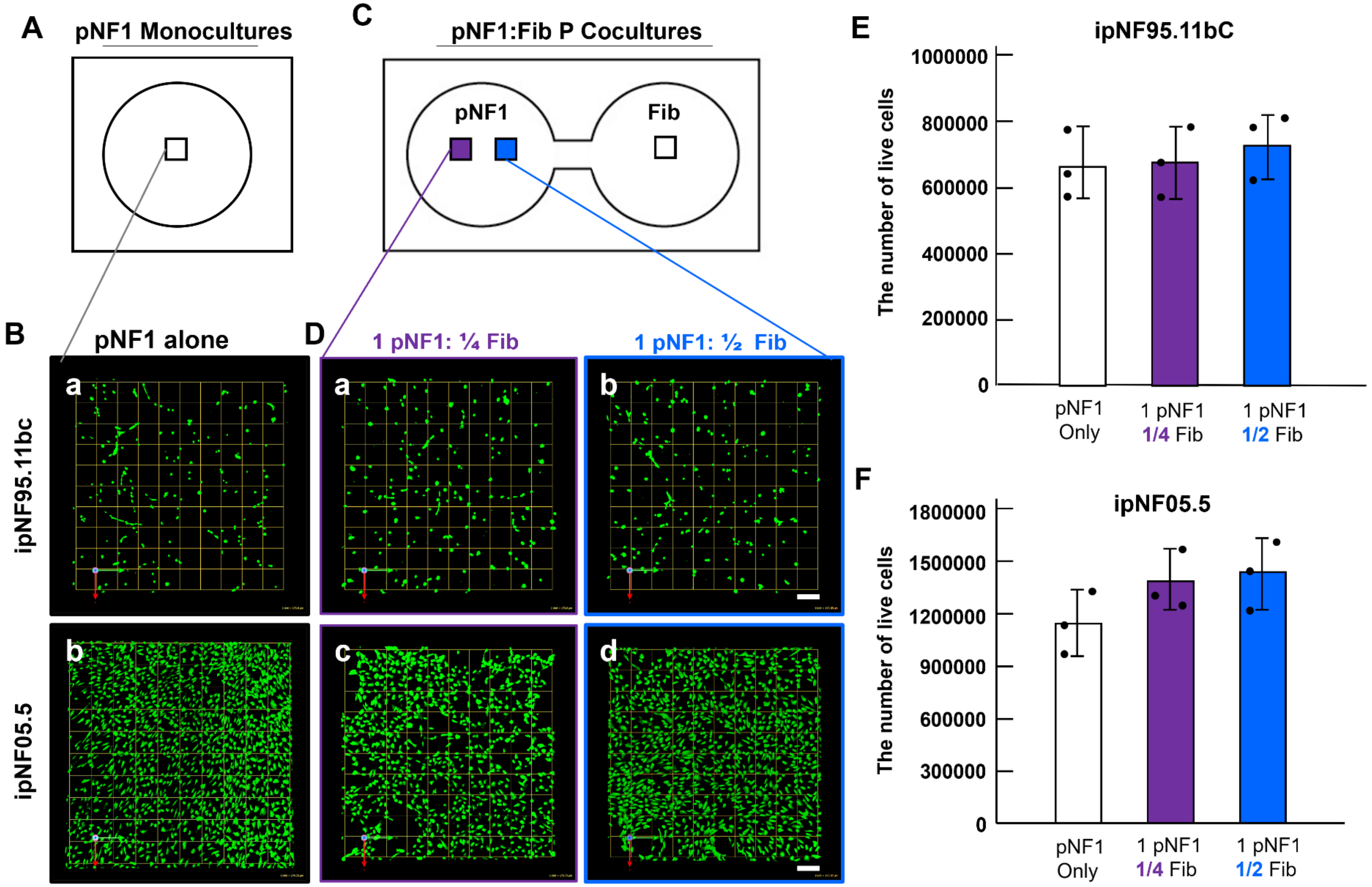

Supplemental Figure S1. Fibroblast-derived secretome induces a minimal increase in pNF1 tumor structure growth in 3D pNF1 cell:Fibroblast parallel cocultures. A, C) Schematics of the TAME chips either with separate wells for 3D pNF1 monocultures (**A**) or with linked wells for 3D pNF1:Fib parallel cocultures (**C**; P Cocultures). Boxes in each well indicate where the images in **B, D** were taken. **B, D)** En face views of 3D reconstructions of 3D pNF1 structures formed by ipNF95.11bC [top row; **B a** and **D a, b**] and ipNF05.5 [bottom; **B b** and **D c, d**] in monocultures (**B**), and in parallel cocultures with fibroblasts at different ratios with selumetinib (**D**). Green signals indicate live cells stained by Calcein AM. Different ratios of pNF1 cells and fibroblasts at 1: ¼ [**D**, left panel (**a, c**)] or 1: ½ [**D**, right (**b, d**)] were used. Images are tiled from 4 contiguous fields; a grid and scale bars, 174 μm. **E, F)** Total number of live pNF1 cells [ipNF95.11bC (**E**) and ipNF05.5 (**F**)] in monocultures (white bars, no treatment) and parallel cocultures (purple, 1pNF: ¼ Fib parallel coculture; blue, 1pNF:½ Fib parallel coculture) was quantified in 3D using Volocity. Data are expressed as mean ± SD (n=3).
